# A Novel Approach to New-Onset Hemiplegic Shoulder Pain With Decreased Range of Motion Using Targeted Diagnostic Nerve Blocks: The ViVe Algorithm

**DOI:** 10.3389/fneur.2021.668370

**Published:** 2021-05-28

**Authors:** John W. Fitterer, Alessandro Picelli, Paul Winston

**Affiliations:** ^1^Canadian Advances in Neuro-Orthopedics for Spasticity Congress, Victoria, BC, Canada; ^2^Section of Physical and Rehabilitation Medicine, Department of Neurosciences, Biomedicine and Movement Sciences, Neuromotor and Cognitive Rehabilitation Research Center, University of Verona, Verona, Italy; ^3^Division of Physical Medicine and Rehabilitation, University of British Columbia, Victoria, BC, Canada

**Keywords:** muscle spasticity, complex regional pain syndromes, bursitis, stroke, rehabilitation

## Abstract

**Introduction:** Hemiplegic shoulder pain (HSP) is the most common pain disorder after stroke with incidence estimates of 30–70% and associated with reductions in function, interference with rehabilitation, and a reduced quality of life. Onset may occur as soon as a week after stroke in 17% of patients. Management of HSP represents a complex treatment pathway with a lack of evidence to support one treatment. The pain has heterogeneous causes. In the acute setting, decreased range of motion in the shoulder can be due to early-onset spasticity, capsular pattern stiffness, glenohumeral pathology, or complex regional pain syndrome (CRPS). As contracture can form in up to 50% of patients after stroke, effective management of the painful shoulder and upper limb with decreased range of motion requires assessment of each possible contributor for effective treatment. The anesthetic diagnostic nerve block (DNB) is known to differentiate spasticity from contracture and other disorders of immobility and can be useful in determining an appropriate treatment pathway.

**Objective:** To create a diagnostic algorithm to differentiate between the causes of HSP in the stiff, painful shoulder in the subacute setting using diagnostic techniques including the Budapest Criteria for CRPS and DNB for spasticity and pain generators.

**Results:** Examination of each joint in the upper extremity with HSP may differentiate each diagnosis with the use of an algorithm. Pain and stiffness isolated to the shoulder may be differentiated as primary shoulder pathology; sensory suprascapular DNB or intra-articular/subacromial injection can assist in differentiating adhesive capsulitis, arthritis, or rotator cuff injury. CRPS may affect the shoulder, elbow, wrist, and hand and can be evaluated with the Budapest Criteria. Spasticity can be differentiated with the use of motor DNB. A combination of these disorders may cause HSP, and the proposed treatment algorithm may offer assistance in selecting a systematic treatment pathway.

## Introduction

Hemiplegic shoulder pain (HSP) is the most common pain disorder after stroke and one of the four most common complications. The estimated incidence ranges from of 30–70% (1–3). HSP is associated with reductions in function, interference with rehabilitation efforts (4), and a reduced quality of life (5). Onset of HSP is rapid, occurring as soon as a week after stroke in 17% of patients (6). While HSP is ubiquitous (1, 7), the management of HSP represents a complex treatment pathway with insufficient evidence supporting one particular treatment (8). It is known that permanent loss of range of motion (ROM) can occur within 3–6 weeks, and according to the 2019 Canadian Stroke Best Practice Recommendations (CSBPR), there is a lack of evidence supporting individual treatments (9).

The pain associated with HSP may be due to heterogeneous causes. In the acute setting, decreased ROM in the shoulder may represent several processes including the early onset of spasticity, capsular pattern stiffness, glenohumeral pathology, or a component of complex regional pain syndrome (CRPS) known as the post-stroke shoulder hand syndrome. Effective management of the HSP with decreased ROM requires assessment of each possible contributor (3). Multiple factors contribute to normal shoulder positioning and function. Proper shoulder positioning involves a stabilized glenohumeral joint (actively and passively), appropriate glenoid fossa angle, and correct scapular and vertebral column alignment (10). The suprascapular nerve is not only vital to motor function, it provides up to 70% of the sensory fibers to the shoulder (11, 12), and pain transmitted by the suprascapular nerve represents another potential underlying cause of HSP (13).

The pathology attributed to HSP includes a variety of neurological and mechanical factors, and effective treatment is heavily influenced by accurate and early detection of these factors. The lack of early recognition of the causative pathology of HSP can lead to worsening limb function and increased pain and may impair the rehabilitation and recovery process, as the pain may peak at up to 4 months (3, 14, 15). Early intervention may reduce the risk and onset of contracture, which can lead to significant impairments and a reduced quality of life (15–17). The incidence of developing at least one contracture in stroke patients within 6 months of their stroke is estimated at 52% (18). Contractures are a source of pain and limited ROM, limit function (18, 19), and require intensive treatment, as stretching has not shown to be clinically effective (20). Patients with contractures and limited volitional control of their shoulder muscles, adduction, or internal rotation can experience maceration, skin dehiscence, impaired axillary hygiene, and difficulty dressing (21, 22). For these patients, relief of the contracture and an improvement of passive function may require surgery (shoulder tenotomies) to release the pectoralis major, latissimus dorsi, teres major, and subscapularis muscles (21).

The use of diagnostic nerve blocks (DNBs) in the assessment of spasticity has gained renewed attention. Sensory DNB may increase ROM and reduce spasticity through the reduction of pain (23, 24), while motor DNB has a more direct impact on temporarily reducing spasticity by paralyzing the targeted nerve branch to a muscle (25). Tardieu and Hariga (26) first described the use of DNB for spasticity in 1964. DNBs have proven to be effective at differentiating between spastic hypertonia, joint stiffness, and contracture (27). DNBs can be used to predict how a patient will respond to local chemodenervation with botulinum toxin (BoNT) or phenol/alcohol. A DNB will cause a spastic muscle to relax to its maximal length, whereas a contracture will have minimal or no change.

Despite the importance of early diagnosis and intervention, few guidelines exist to systematically direct early care. There is a lack of validated and reliable instruments to assess pain associated with arm spasticity (28). Management of HSP has been proposed in many review articles, which includes management of the flaccid or subluxed shoulder. Guidelines, such as the CSBPR, present the evidence for treating the individual pathologies that result in HSP; however, there is no current clinical pathway to assist the clinician in identifying the causative pathologies and how to treat the contribution of each pathologic process. The new onset of HSP presents additional diagnostic challenges due to patient-specific factors, such as decreased levels of arousal, language disorders, weakness, limited exercise tolerance, medical comorbidities, and sensory and visual–spatial neglect. Furthermore, HSP with reduced ROM may not be isolated and may be accompanied by stiffness, pain, and reduced ROM in the distal upper extremity joints. Thus, the ability to accurately differentiate between the causes of painful reduced ROM, including isolated shoulder pain, CRPS, spasticity, and contracture requires a method to differentiate the etiologies.

In the absence of an established clinical pathway, we have drawn on our own use of the clinical evidence, such as prednisone for the treatment of CRPS (29), the use of motor DNB for spasticity assessment, suprascapular DNB for isolated shoulder pain (13, 30, 31), and early intervention for spasticity (32). We propose an algorithm to assist in the assessment and management of new-onset HSP in shoulders with stiffness and a reduced ROM built from our own clinical experience in treating this condition. We propose a clinical and interventional pathway, the ViVe Algorithm, to assess each component and guide the treatment of each condition.

## Methods

The development of the ViVe Algorithm involved a pathway created from knowledge gained through routine clinical care of HSP at one site in Canada and one in Italy. Both senior authors have extensive clinical experience in treating new-onset HSP. This includes international collaboration in early assessment and treatment of spasticity (32). Both clinicians have experience and publications in ultrasound-guided DNB. Additionally, the authors have a routine clinical practice and publications in either suprascapular DNB or the assessment and treatment of CRPS. The algorithm is thus a joint collaboration of our routine care that recognizes the clinical challenges in the treatment of new-onset HSP. Development of the algorithm required the involvement of the multidisciplinary rehabilitation teams. It is essential to underscore that nursing, physical, and occupational therapists are key players who continually assess and report on the development of HSP. The foundation for the algorithm is in the assessment and feedback from the therapy teams that report on pain, ROM, and presence of symptoms of CRPS, spasticity, and contracture. In turn, the components of the ViVe Algorithm were created based on the signs and symptoms identified by the therapy teams and the diagnostic uncertainties that arose in consultation with the physician/therapy teams. In contrast to the traditional approach, our algorithm was created through collaborative assessment of each joint in the upper extremity to assess the etiology of its limited and painful ROM. The DNB facilitates that differentiation.

A literature review of the anatomy, assessment, and treatment of isolated shoulder pain, CPRS, and spasticity was conducted. Sample illustrative case studies were created to illustrate the pathways of the algorithm, with a guide to the clinical examination and diagnostic procedures.

When it is unclear if decreased ROM is due to isolated shoulder pain, CRPS, or spastic muscle overactivity, DNBs are implemented. Suprascapular sensory nerve blocks are performed for isolated shoulder pain along with intra-articular injections (**Figure 2**).

The presence of CRPS is assessed using the Budapest Criteria with guidance from the CSBPR. Treatment with prednisone was offered for the CRPS using recommended doses (29, 33).

To assess the contribution of spastic muscle overactivity, we implemented the lateral pectoral nerve (LPN) block as the primary DNB for the adducted shoulder (**Figure 3**). Musculocutaneous (MSCN) DNBs to the brachialis, biceps, and the radial nerve to the brachioradialis are utilized for the spastic flexed elbow (25). Radial and median nerve blocks are for the wrist and fingers.

The DNB is performed by the infiltration of small volumes (1–4 ml) of a 2% lidocaine around a nerve or nerve fascicle to select the most responsible muscles in a spastic deformity (25, 34, 35). To assist localization, we recommend that they are performed in combination with e-stimulation and ultrasound (30, 31).

Isolated pain in the shoulder or other joints is assessed, and other causes of joint inflammation, arthropathy, or heterotopic ossification are ruled out by clinical judgment and testing, including diagnostic imaging of the shoulder as indicated.

The patient's active range of motion (AROM) and passive range of motion (PROM) are evaluated for spasticity and measured using the Modified Ashworth Scale (MAS) and Modified Tardieu Scale (MTS). For CRPS, the presence or absence of the signs and symptoms of the Budapest Criteria is utilized.

## Results

**Figure 1:**
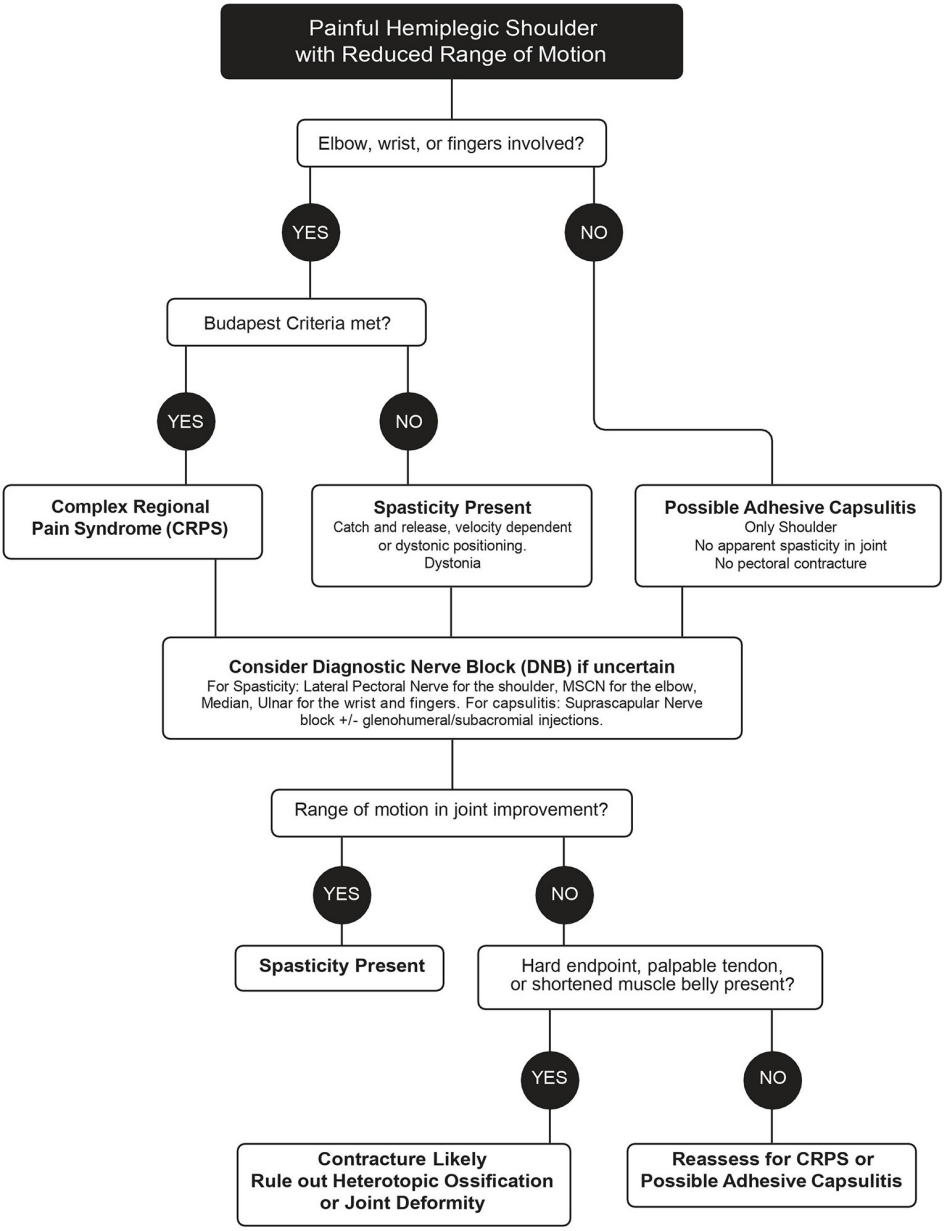
ViVe treatment algorithm.

The ViVe Algorithm was developed to allow characterization of the stiff hemiplegic shoulder with and without involvement of the distal upper extremity joints (Figure 1). It was designed for the post-acute state when spasticity, CRPS, and capsulitis typically first develop. When HSP is first identified, the algorithm next asks if elbow, wrist, or fingers are involved. If not involved, isolated shoulder pain, such as capsulitis is suspected. In the absence of apparent spasticity or pectoral contracture, treatment is for isolated shoulder pain, including capsulitis; suprascapular DNB and intra-articular injections are offered. If capsulitis is uncertain, then a DNB of the LPN to assess for spasticity is performed. If ROM improves, then spasticity is presumed present and treated as such. If not, capsulitis is again suspected, but joint pathology, heterotopic ossification, and contracture should be ruled out through medical imaging and palpation of contracted tendons.

If the elbow, wrist, or fingers are involved, an assessment with the Budapest Criteria for CRPS is performed as well as an assessment for the presence of spasticity. If the criteria are met, treatment with oral prednisone is recommended initially. If differentiation between spasticity and CRPS is uncertain, a DNB of the MSCN and/or radial nerves is performed for the elbow and potentially the median and/or ulnar for the wrist and fingers. Significant improvements in ROM after the DNBs suggest that the cause of the restricted ROM is spasticity. If there is no improvement, early contracture, joint deformity, and heterotopic ossification are to be ruled out. The algorithm is designed to be revisited after each treatment.

### Illustrative Composite Cases

#### Case 1

A patient in their 80s presented with dense right hemiplegia due to a left frontal subcortical infarct. The right arm was flaccid initially. There was no history of prior shoulder pain. At 2 weeks post-stroke assessment, the algorithm found reduced painful PROM involving the shoulder, elbow, wrist, and fingers. At 3 weeks, the therapy team noted that the left hand and wrist were swollen and painful with brawny skin; they were shiny and warm. The limb met the Budapest Criteria for CRPS in the wrist and hand with minimal joint movement in the painful wrist and fingers. The presence of spasticity was assessed. The elbow had reduced extension with an initial fast spastic catch or V3 at 90° and maximal slow ROM or V1 of 160° on the MTS and a MAS grade of 2. The shoulder abduction was up to 90°, limited by pain, thus spasticity could not be properly assessed, though a taut band was felt in the pectoralis muscles with abduction. A decision to first treat the CRPS was made due to the whole limb involvement. Here, 60 mg of prednisone was started with a daily taper (33). Within 5 days, the swelling in the hand resolved, passive manipulation was possible due to reduced pain. The temperature and discoloration normalized. There was mild stiffness, but no spasticity in the hand or wrist. The algorithm was re-implemented to assess the ongoing elbow and shoulder reduced ROM. The MAS remained 2 at the elbow and was now 2 with shoulder abduction. A DNB was performed to both the LPN and MSCN branch to the brachialis. This resulted in full PROM in both elbow and shoulder and a drop in MAS grade to 1+. The cause of the reduced ROM was determined to be due to spasticity. Then, 150 units of onabotulinum A (BoNT) were injected (50 units to the pectoralis, 60 units to the brachialis, and 40 units to the brachioradialis with ultrasound guidance). At 2 weeks' follow-up, there was no reported pain and increased PROM in the wrist and fingers. At 3 months, PROM was full in the elbow and shoulder with some active antigravity movements. There was no active movement in the fingers, but they were pain-free with full ROM.

#### Case 2

A patient in their 70s suffered a large subarachnoid bleed. There was no known prior history of shoulder pain. At 3 weeks, the examination found a painful capsular pattern to the shoulder, with passive abduction to 70°. A MAS grade of 2 was noted in the elbow flexors, which had a V3 of 110° and V1 of 170°. There was a spastic catch and release in the limited shoulder ROM, with a taut band in the pectoralis muscle noted, but the presence of pain made it challenging to measure. There were no features of CRPS. Using the algorithm, a diagnosis of isolated shoulder pain, with early capsulitis, was made in the presence of spasticity. A glenohumeral cortisone injection with 40 mg of Depo-Medrol and 4 cc of 2% lidocaine with ultrasound guidance was performed, as well as a guided suprascapular DNB with 2 cc of lidocaine. At 15 min, the shoulder pain was significantly diminished, and PROM had improved by 30°, but spasticity was unmasked to reveal MAS of 2 in the shoulder. On day 4, the pain was improved but spasticity remained. Using the algorithm, 150 units of BoNT were administered to the elbow flexors and shoulder adductors (brachialis, brachioradialis, and pectoralis muscles). Muscle tone continually improved over the course of 1 month, and there was full PROM of the shoulder and elbow with MAS grade of 1. No further treatment was required long-term.

#### Case 3

A patient in their 50s presented with left hemiplegia due to a right frontal lobe infarct. Within 5 days, there was left arm weakness with spasticity of MAS grade of 3 isolated to only the elbow flexors. There was no reported shoulder pain. There were active movements with gravity eliminated in the elbow and strong finger flexors. Treatment was offered with a total of 125 units BoNT to the brachialis and brachioradialis. At 1 month, ROM at the elbow had greatly improved, both actively and passively with a MAS of 1, with no V3, and a full V1 of 180°. At 3 months, as an outpatient, there was now a painful stiffness in the shoulder, limiting abduction and external rotation to <90°. Using the algorithm, it was notable that elbow flexor tone was normal, and the wrist and hand remained unaffected. No taut band was felt in the pectoralis muscle when the shoulder was passively abducted. There were no features of CRPS. It remained unclear if this was isolated shoulder pain or spasticity. A DNB of the LPN was performed. The pectoral muscles went flaccid post-injection, but no change in ROM occurred. Using the algorithm, a glenohumeral cortisone injection with a suprascapular DNB was performed, and the pain immediately improved, but minimal improvement in shoulder ROM was noted. An intra-articular cortisone injection was declined by the patient. A diagnosis of adhesive capsulitis was made. At 1 year post-stroke, active movements in the whole limb were graded from 4 to 5 (MRC), and spasticity was not detectable in the limb. The left shoulder ROM was improved with little pain, but there remained a mild capsular restriction.

## Discussion

The ideal management of HSP remains elusive. Patients typically receive focal intensive therapies in the initial months after a stroke, along with inpatient and outpatient therapies. A 2021 review found that 9.4% of patients with paresis will develop severe or disabling spasticity (36). Hefter et al. (37) noted that four of the five most common spasticity patterns in the upper extremity include adduction and internal rotation of the shoulder. Kwah et al. (18) demonstrated that of 200 consecutive patients, 25% of all stroke patients and 38% of moderate to severe stroke patients developed a shoulder contracture within 6 months (22 and 35% for elbows, 13 and 29% for wrists, respectively). Early intervention to prevent contracture development and disuse is the goal of post-stroke spasticity (PSS) care. Deltombe et al. (35) demonstrated the role of DNBs in differentiating spasticity from contracture in the foot and offered an elegant algorithm to guide the clinician. The post-acute HSP offers an additional diagnostic challenge due to frequency of CRPS and capsulitis in addition to the spasticity. Accurate assessment is additionally complicated by the possibility that CRPS, adhesive capsulitis, and spasticity may all be actively developing. Hence, the need for an algorithm to reflect this time dependence and heterogeneity.

The shoulder hand syndrome after stroke is a well-described variant of CRPS (38, 39), with an estimated incidence of up to 48.8% in the first 28 weeks (14). Risk factors include motor disability and trauma related to altered shoulder biomechanics. The two major types of CRPS (CRPS types I and II) are characterized by the presence or absence of an identifiable nerve injury. CRPS type I is not limited to an individual peripheral nerve and is associated with edema, skin blood flow abnormalities, and sudomotor activity in the region of the pain in the form of both allodynia and hyperalgesia. CRPS type II occurs after injury to a nerve or a major nerve branch innervating a particular region and causes a characteristic burning sensation in addition to allodynia and hyperpathia within the region. Clinical diagnosis is assisted by the patient's medical history and assessed by clinical examination using the Budapest Criteria (40, 41). Kalita et al. (42) proposed an assessment of post-stroke CRPS based on the work of Braus et al. (43).

The CSBPR offer that the diagnosis should be based on clinical findings including pain and tenderness of the metacarpophalangeal and proximal interphalangeal joints and can be associated with edema over the dorsum of the fingers, trophic skin changes, hyperesthesia, and limited ROM as level C evidence (9). When clinical diagnosis is unclear, the most commonly used imaging modalities to assist diagnosis are triple-phase bone scans, which detect the characteristic increased periarticular uptake in the distal joints (44, 45).

Altas et al. (46) examined the risk factors and clinical treatment parameters associated with post-stroke CRPS and concluded that the nociceptive and neuropathic pain etiologies encompassed by CRPS complicate treatment, and optimal treatment often requires a combination of treatment modalities. These can include pharmacological, orthotic, biomechanical, and electrophysiological therapies. In a review of the current CRPS literature, Van Eijs et al. (47) emphasized prioritization of pharmacological pain management and physical rehabilitation of limb function, noting they should be started as early as possible, while interventional pain management techniques are only considered when there is no improvement to limb function or pain.

A number of analgesic and anti-inflammatory pharmacological treatments have been assessed for efficacy in managing CRPS, including bisphosphonates, calcitonin, and corticosteroids. Corticosteroids are commonly used to treat CRPS post-stroke; a 1994 study showed 31 out of 34 post-stoke patients who developed CRPS achieved near-total relief within an average of 10 days following corticosteroids (43). Two studies by Kalita et al. (29, 42) demonstrated the viability of prednisolone (40 mg), as 89.7% of patients with post-stroke CRPS experienced an improvement in their symptoms following 1–2 months of treatment. Continuation of a lower dose of prednisolone (10 mg) for an additional 2 more months resulted in no recurrence of CRPS and continual improvement, whereas 50% had a recurrence when prednisolone treatment was stopped. Patients who discontinued treatment after 2 weeks had deterioration at 1 month and required reinstitution of prednisolone, following which 77% of them saw CRPS improvement within the next month, emphasizing the importance of monitoring the patients for resolution (29). The CSBPR affords prednisone as a treatment of CRPS as Level B evidence (9). The presence of CRPS severely compromises clinical assessment of spastic muscle overactivity due to the effects of pain with the movement of multiple joints; therefore, CRPS should be rapidly assessed and treated.

Post-stroke HSP has been attributed to rotator cuff disease, adhesive capsulitis, and spasticity subluxation (1, 7). It is important to rule out preexisting causes or common causes such as rotator cuff tendinopathy or osteoarthritis, as well as acquired plexus or lower motor traction injuries from the handling of the hemiplegic shoulder (48, 49). There is evidence showing that swollen suprascapular nerves might be related to painful shoulders in the aging population (50). Resistance to movement commonly coincides most with both capsulitis and spastic muscle overactivity.

**Figure 2:**
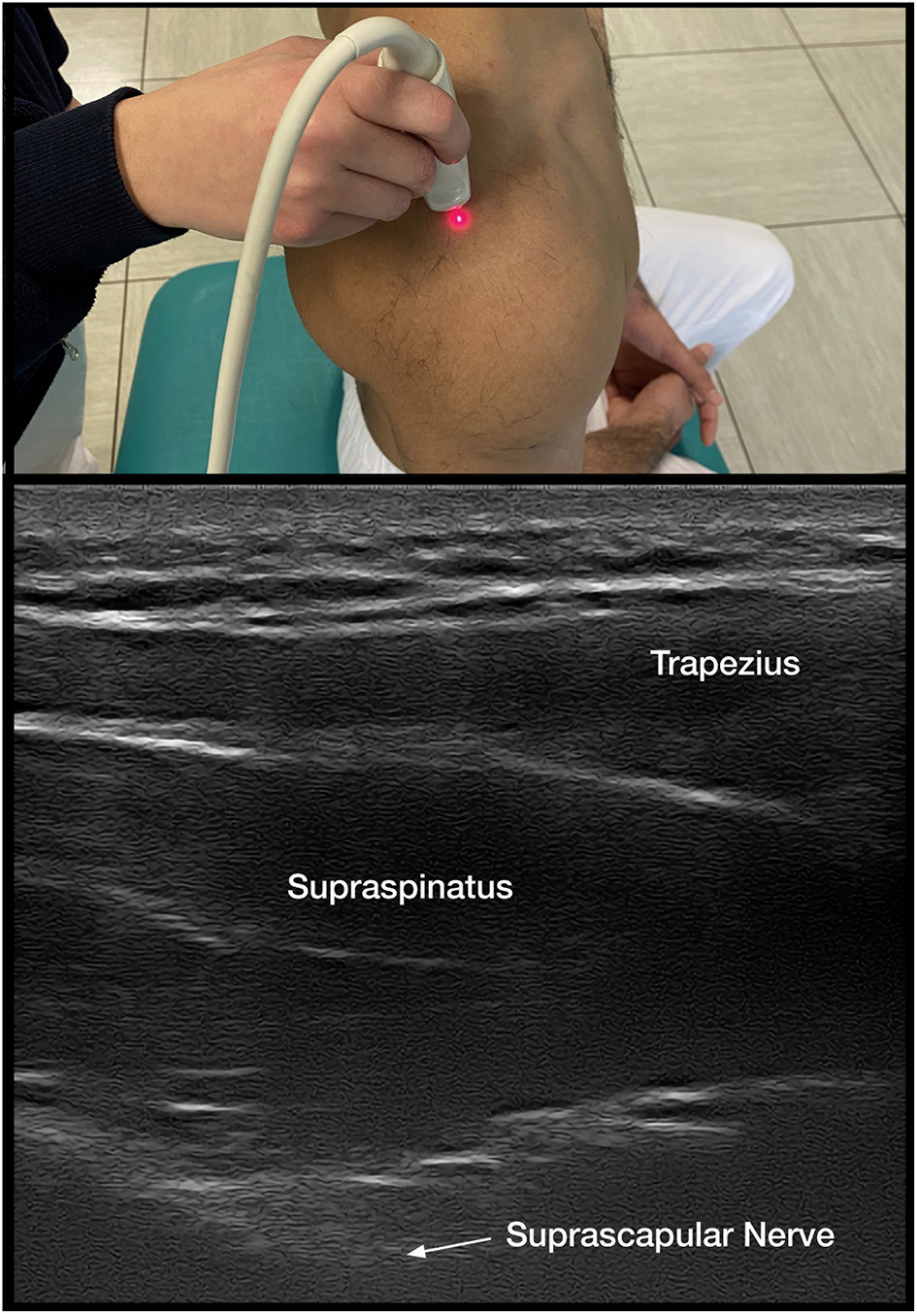
Suprascapular nerve block.

Both the suprascapular DNB and intra-articular corticosteroids are shown to decrease pain and improve ROM in HSP (1, 7, 13, 51, 52) (Figure 2). Either intervention is readily accessible to clinicians providing care and may assist in distinguishing the contributions of pain vs. spastic muscle overactivity. Intra-articular injections with anesthetics and DNB allow one to immediately assess if capsular or joint pain is contributing to decreased ROM, as PROM and, if possibly, AROM are reassessed within minutes. A successful reduction of pain with a DNB also allows the implementation of longer-lasting nerve procedures with techniques that include phenol (53, 54) or pulsed radiofrequency (52, 55).

Upper extremity spastic muscle overactivity has been identified as one of the most common causes of HSP and may appear in the acute phase (56). Regardless of the pathology, a review found that HSP is higher than normal in patients with PSS (57). HSP patients showed significantly more spasticity in affected limbs compared to limbs that are pain-free (58). Reported prevalence of PSS, most commonly assessed using the MAS (59), ranges from 4 to 27% during the first 6 weeks after stroke (60).

Early treatment includes physical modalities, bracing, oral agents, and focal chemodenervation with BoNT or phenol/alcohol. While few studies have addressed early treatment with BoNT, the few available note that a trend of successful early treatment is possible with reduced doses (61–63). The most commonly targeted muscles for spasticity of the shoulder include the pectoralis and subscapularis muscles (53, 64–66), although consensus on which muscle to treat with BoNT varies widely (66). Treatments of PSS related to HSP are centered around improving arm abduction and external rotation. In the presence of pain, the assessment of spastic muscle overactivity may be compromised due to inability to completely range a joint, thus as addressed previously, pain reduction is a key first step and clinical assessment and treatment of CRPS and isolated shoulder pain should precede assessment of spastic muscle overactivity.

The development of PSS related to HSP may impair ROM. The pectoralis and subscapularis muscles contribute largely to the abduction and internal rotation of the shoulder. The pectoralis major muscle (PMM) is primarily innervated by branches of the brachial plexus; the MPN's role is supplementary, whereas the LPN's increased thickness and muscle innervation make it vital to proper function (67), and injury can result in total denervation, atrophy, and fibrosis of the PMM (68). Spasticity in the subscapularis muscles can limit shoulder abduction, flexion, and external rotation (23). Scapular rotation can also be impaired due to increased tone in the muscles attached to the scapular surface (latissimus dorsi, levator scapulae, rhomboid). These muscles surround the scapula and assist with abduction and the external and internal rotation of the glenohumeral joint.

**Figure 3:**
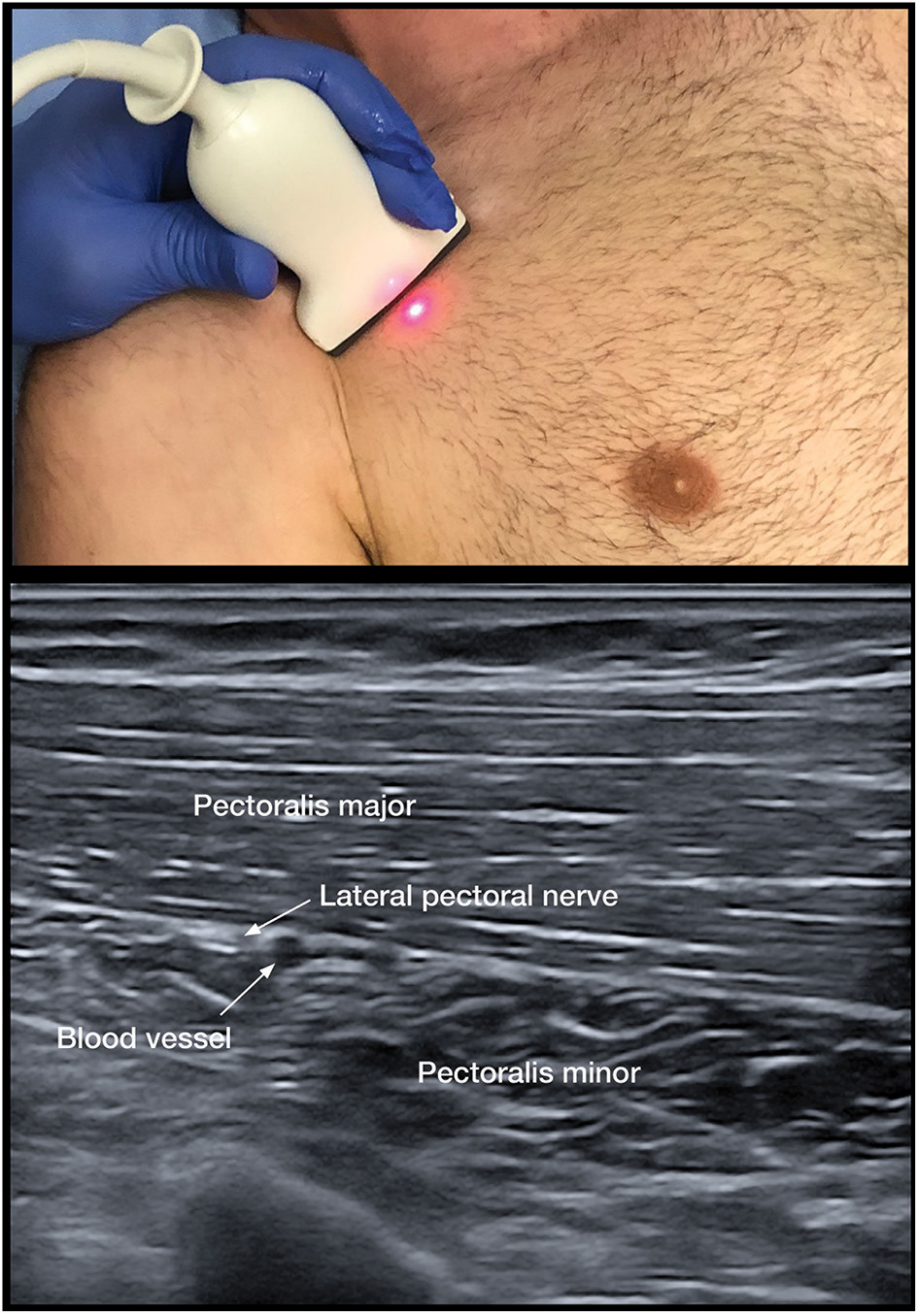
Lateral pectoral nerve block.

As the adducted shoulder is a common painful pattern, pectoral DNB remains a key option to distinguish between spastic muscle overactivity and capsulitis. The PMM is the dominant and largest muscle with clavicular, sternal, and abdominal portions (69). The course of the nerve has been found to be consistent in anatomical studies that demonstrate that the LPN was found to course alongside the pectoral branches of the thoracoacromial blood vessels on the undersurface of the PMM in 100 consecutive patients (68) (Figure 3). The LPN was shown to innervate both heads of the PMM (32). Targeting the LPN may also have an effect on the pectoralis minor muscle, as it gives innervation to this muscle through the communicating ansa pectoralis (70). We have used the LPN in our algorithm, as the MPN is less consistent and deeper to access and has numerous branches (69). Unlike, the pectoral muscles, the subscapularis cannot be palpated and has numerous innervations; thus, DNB is not practical (71). The thoracodorsal nerve is highly consistent in its course. The nerve can be targeted after it enters the latissimus dorsi at a point midway along the thorax (72).

For the elbow, the recommended targets for nerve blocks are the branches of the MSCN nerve to the brachialis and biceps and the radial nerve to the brachioradialis (25, 31). For the wrist and fingers, median and ulnar nerve DNBs are performed.

Changes after motor DNB are documented by measuring changes to the fast catch V3 and slow stretch V1 ROM on the MTS (73). Filipetti and Decq (74) demonstrated the value of incorporating DNBs into the treatment approach of a cohort of 566 patients. Functional assessment was carried out using the MTS and MAS before and after DNB administration and determined that DNBs assisted in determining the relative contribution of the muscle or muscles contributing to overactivity and spasticity and muscle shortening leading to pathologic posture. They concluded that the method's ability in predicting new functional balance and treatment effectiveness is particularly valuable, and DNBs represent a necessary stage to patient assessment (74).

This algorithm has numerous limitations. The CSBPR notes the lack of evidence to evaluate and support any treatment pathway for HSP. Accepted outcomes for CRPS and spasticity are equally elusive, and there is no accepted universal outcome yet to validate the ViVe Algorithm. In the acute to subacute phase, weakness, flaccidity, visual and sensory neglect, and cognitive and language deficits preclude the use of most outcome tools. Lastly, the DNB is not universally practiced and requires knowledge of anatomy and technique. We note that the most up-to-date guide on DNB determined that Ultrasound (US) has not yet been sufficiently noted be superior to anatomical landmark, based on a paucity of literature (27). This literature is growing (30, 34, 75).

## Conclusion

We believe this to be the first proposed algorithm to assess the early-phase HSP based on the presentation of a painful limb with reduced ROM, to distinguish from cases of flaccidity and subluxation. This algorithm is a proposal to guide clinicians into selecting the most appropriate diagnosis of the etiology of pain and reduction in ROM. Each of the conditions, CRPS, capsulitis, and spasticity, has existing treatment protocols that can used alone or in combination. The goal of the algorithm is to ultimately prevent irreversible contracture formation and pain from impairing recovery in the subacute phase when patients typically receive their most intense phase of rehabilitation. The ViVe Algorithm allows for collaboration between physiotherapists, occupational therapists, and spasticity physicians to develop a treatment pathway that allows all providers to produce optimal medical interventions.

## Data Availability Statement

The original contributions presented in the study are included in the article/Supplementary Material, further inquiries can be directed to the corresponding author/s.

## Author Contributions

PW was responsible for the initial concept to create this paper and algorithm and created the illustrative case examples. JF performed the literature review and manuscript preparation. AP offered guidance, expert opinion, and contributed to the development of the algorithm and the manuscript. All authors contributed to the article and approved the submitted version.

## Conflict of Interest

The authors declare that the research was conducted in the absence of any commercial or financial relationships that could be construed as a potential conflict of interest.
